# Electroacupuncture for chemotherapy-induced peripheral neuropathy: study protocol for a pilot multicentre randomized, patient-assessor-blinded, controlled trial

**DOI:** 10.1186/1745-6215-14-254

**Published:** 2013-08-14

**Authors:** Joo-Hee Kim, Eun-Jung Kim, Byung-Kwan Seo, Sanghun Lee, Seunghoon Lee, So-Young Jung, Min-Hee Lee, Ae-Ran Kim, Hyo-Ju Park, Mi-Suk Shin, Sun-Mi Choi

**Affiliations:** 1 Acupuncture, Moxibustion & Meridian Research Group, Medical Research Division, Korea Institute of Oriental Medicine, Daejeon, South Korea; 2 Department of Acupuncture & Moxibustion, College of Korean Medicine, Kyung Hee University, Seoul, South Korea; 3 College of Korean Medicine, Dongguk University, Gyeongju, South Korea; 4 Department of medical consilence, Graduate school, Dankook University, Gyeonggi-do, South Korea

**Keywords:** Chemotherapy-induced peripheral neuropathy, Electroacupuncture, Effect, Safety, Clinical research protocol

## Abstract

**Background:**

Chemotherapy-induced peripheral neuropathy (CIPN) is the main dose-limiting side effect of neurotoxic chemotherapeutic agents. CIPN can lead not only to loss of physical function, difficulties in activities of daily living (ADLs), and decreased quality of life, but also to dose reduction, delay or even cessation of treatment. Currently, there are few proven effective treatments for CIPN. This randomized controlled clinical trial is designed to evaluate the effects and safety of electroacupuncture (EA) for patients with CIPN.

**Methods/design:**

This is a multicenter, two-armed, parallel-design, patient-assessor-blinded, randomized, sham-controlled clinical trial. Forty eligible patients with CIPN will be randomized in a ratio of 1:1 to the EA or sham EA arms. During the treatment phase, patients will undergo eight sessions of *verum* EA or sham EA twice weekly for four weeks, and then will be followed-up for eight weeks. Electrical stimulation in the EA group will consist of a mixed frequency of 2/120 Hz and 80% of bearable intensity. Sham EA will be applied to non-acupoints, with shallow needle insertion and no current. All outcomes and analyses of results will be assessed by researchers blinded to treatment allocation. The effects of EA on CIPN will be evaluated according to both subjective and objective outcome measures. The primary outcome measure will be the European Organization for Research and Treatment of Cancer (EORTC) quality of life questionnaire to assess CIPN (QLQ-CIPN20). The secondary outcome measures will be the results on the numerical rating scale, the Semmes-Weinstein monofilament test, the nerve conduction study, and the EORTC QLQ-C30, as well as the patient’s global impression of change and adverse events. Safety will be assessed at each visit.

**Discussion:**

The results of this on-going study will provide clinical evidence for the effects and safety of EA for CIPN compared with sham EA.

**Trial registration:**

Clinical Research Information Service: KCT0000506

## Background

Chemotherapy-induced peripheral neuropathy (CIPN) is defined as damage to the peripheral nervous system induced by neurotoxic chemotherapeutic agents, including platinum compounds such as oxaliplatin and cisplatin, taxanes such as paclitaxel and docetaxel, and vinca alkaloids like vincristine [1,2]. Although the symptoms of CIPN vary according to the type and severity of the motor, sensory, and autonomic nerves that are affected, most symptoms of CIPN are sensory and include numbness, tingling, and shooting and burning pains in the toes and fingers, progressing proximally in a typical ‘glove and stocking’ distribution. Furthermore, CIPN in most patients is only partially reversible and can persist long after treatment is terminated [3]. CIPN is one of the major dose-limiting side effects of chemotherapy and can lead not only to loss of physical function, difficulties in activities of daily living (ADLs), and decreased quality of life (QOL), but also to dose reduction or delay or even cessation of treatment [4,5].

Dose and treatment schedule modifications of neurotoxic chemotherapy are currently the primary methods used for the non-pharmacological management of CIPN [3]. Alternative dosing regimens and treatment modification schemes have been found to be effective in reducing the incidence and/or severity of CIPN [6,7]. These strategies, however, can potentially affect tumor response and disease progression. Several pharmacologic agents useful in treating peripheral neuropathy caused by other conditions such as diabetes, have been investigated as treatment for CIPN; these include anticonvulsants such as gabapentin and lamotrigine, and tricyclic antidepressants. To date, however, no agent has proven effective in the treatment or mitigation of CIPN [8,9], underscoring the need for the development of novel treatments.

Acupuncture is a popular and safe treatment intervention used to manage various conditions including peripheral neuropathy. Electroacupuncture (EA) has been found to be effective in treating diabetic peripheral neuropathy [10], neuropathic pain including post-herpetic neuralgia and post-traumatic neuropathy [11], and human immunodeficiency virus (HIV)-related peripheral neuropathy [12]. Clinical trials in patients with CIPN have shown that acupuncture/EA alleviated symptoms [13-17] and improved nerve conduction study (NCS) results [18], suggesting that EA may be an effective treatment for CIPN without causing adverse effects. Most prior studies, however, were case reports, case series, or clinical trials lacking a control group, resulting in a high risk of bias. A recent Cochrane Review on acupuncture for cancer pain included three randomized controlled trials (RCTs), only one of which was of high methodological quality [19]. In addition, few RCTs have evaluated EA for CIPN, underscoring the need for well-designed robust clinical trials to provide evidence for EA in treating CIPN.

### Aim of the study

The aim of this study is to test the feasibility of a RCT to investigate effectiveness and safety of EA, compared with sham EA, in the treatment of CIPN. We will evaluate whether EA improves symptoms and functioning, cutaneous sensation, nerve conduction, and QOL. All adverse events will also be assessed.

The results of this study will provide evidence for the feasibility of this clinical trial design and also yield data to determine the appropriate sample size for future large-scale RCTs of EA in cancer patients with neuropathy.

## Methods/design

### Study design and ethics

**Figure 1:**
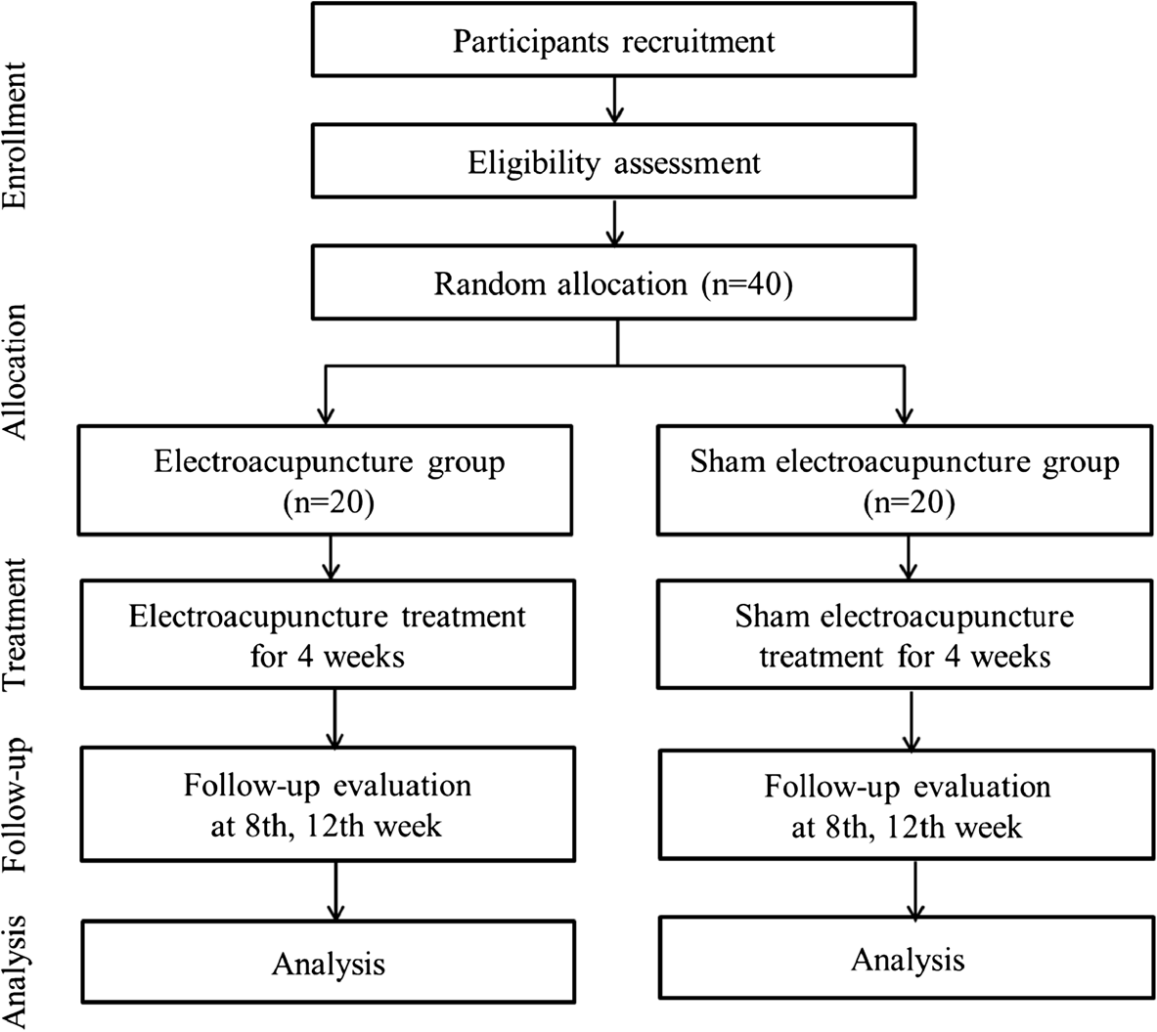
Flowchart showing the steps in patient recruitment, treatment, and analysis.

This is a patient-assessor-blinded, randomized, sham-controlled clinical trial with two parallel arms. The trial will be conducted at two clinical research centers in Korea; Kyung Hee University Hospital at Gangdong and Dongguk University Ilsan Oriental Hospital, in accordance with the Declaration of Helsinki and the Guidelines for Good Clinical Practice. This research protocol has been reviewed and approved by the institutional review boards (IRB) of each trial center (KHNMC-OH-IRB 2012-007 and 2012-01). Written informed consent will be obtained from all study participants prior to enrollment. Eligible participants will be randomized in a ratio of 1:1 to the EA or sham EA arm and receive treatment for four weeks. Patients will be followed-up for two months thereafter (Figure 1). Participants will be evaluated and results will be analyzed by professionals blinded to group allocation. This protocol has been registered with the ‘Clinical Research Information Service’ of the Republic of Korea, a registry in the WHO Registry Network.

### Participants

#### Inclusion criteria

Patients will be eligible if they are aged 18 years or older, have received or are currently receiving neurotoxic chemotherapy, have experienced symptoms of chemotherapy-induced peripheral neuropathy for ≥ one month, and have scores ≥ 4 on 10 on the numerical rating scale (NRS). Eligibility criteria will also include no use of medication to prevent or treat neuropathy for two weeks before screening or stability of medication to prevent or treat neuropathy for two months before screening; an Eastern Cooperative Oncology Group (ECOG) performance status (PS) of 0 to 2; life expectancy of ≥ six months; and provision of written informed consent.

Stable medication is defined as no change in the type and dose of medication to prevent or treat neuropathy for > two months before enrollment. Patients receiving any of those drugs will have to remain on the same medications and doses throughout the study period. Patients requiring a change in the type and dosage of medication will be withdrawn from the study.

#### Exclusion criteria

Exclusion criteria are pre-existing peripheral neuropathy or a history of peripheral neuropathy due to any cause other than chemotherapy (for example, diabetes, peripheral vascular disease, HIV, alcohol, toxins, hereditary factors and nerve compression injuries); known hypersensitivity to acupuncture or inability to cooperate with the acupuncture procedure; treatment with traditional Korean medical protocols, including acupuncture, moxibustion, cupping or herbal medicine, for peripheral neuropathy during one month before enrollment; cardiovascular disorder including arrhythmia or use of a pacemaker; pregnancy or potential pregnancy and nursing; and unwillingness to comply with this study protocol.

### Randomization and allocation concealment

A total of 40 participants who meet the eligibility criteria will be randomised in a ratio of 1:1 to the EA or sham EA group. In this trial, constant exposure to neurotoxic agents and concomitant use of neuropathy medications during the study are variables that present potential confounding effects for the study results. Therefore, this randomization will be stratified according to the receipt of on-going chemotherapy during enrollment or completion of therapy and according to the use of medication for neuropathy. Patients will be randomized using a computer-generated random allocation sequence through the stratified block randomization method of SAS version 9.1.3 (SAS Institute Inc., Cary, NC, USA) by a statistician (MHL) with no clinical involvement in this trial. Sequentially numbered opaque sealed envelopes containing the randomization assignments will be delivered to the clinical trial center. Allocation concealment will not be broken until the final data analysis report is completed.

### Blinding

In this acupuncture research, it is not feasible to conceal allocation from the practitioners, and the practitioners will be aware of the allocation of each patient. However, treatment and assessment will be performed independently, and the practitioners will not be involved in assessing treatment outcomes. The subjects, the outcome assessors, and the statistician performing the data analyses will be blinded to treatment allocation throughout the study. Patient blinding will be assessed after the end of treatment.

### Intervention

#### Electroacupuncture group

EA will be administered twice weekly for four weeks. Bilateral LI4, TE3, GV39, GV41, SP6, LR3, *Ba Feng*, and *Ba Xie* will be used for acupuncture treatment. The acupuncture points were selected by the consensus of an expert committee composed of professors and researchers who specialize in traditional Korean medicine, on the basis of a literature review [13,14,16-18] and a textbook [20].

Single-use, sterile, filiform acupuncture needles, 0.25 mm in diameter and 40 mm in length (Dongbang Co., Seoul, Korea), will be inserted and stimulated manually, but no specific *de qi* sensation will be provoked because the sensitivity of acupuncture points may vary, especially in patients with chemotherapy-induced peripheral neuropathy. A battery-operated EA device (PG-306 pulse generator, Suzuki Iryoki, Tokyo, Japan) will be connected to the bodies of needles at the acupuncture points. Electrical stimulation will be delivered for 30 minutes at alternating frequencies of 2 and 120 Hz, at an intensity of 80% of each patient’s maximum tolerance.

#### Sham electroacupuncture group

Participants allocated to the sham EA group will receive the sham EA treatment twice weekly for four weeks. The total number of visits and acupuncture needles will be identical to those of the *verum* EA group. The selected sham points are as follows: bilaterally at one point at the middle of the biceps brachii muscle belly and one point at 2 cm vertically below the middle, one point at the middle of the brachioradialis muscle belly and points at 1.5 cm, 3 cm, and 4.5 cm vertically below the middle, four points above the upper part of the patella, one point at the upper third above the medial part of the tibia and points at 1.5 cm, 3 cm, and 4.5 cm vertically below the upper third.

The same type of needles will be shallowly inserted into the sham points with no manipulation for *de qi* and will be connected to a deactivated EA device. No current will be applied but the apparatus will emit the same beeping sound and flashing light continuously for 30 minutes. Sham treatment will be performed by the same doctors who perform the *verum* treatment.

#### Education of practitioners

All EA and sham EA treatments will be performed by doctors of Korean medicine who have been certified by the Korean Ministry of Health and Welfare, have at least three years of clinical experience, and have received more than six years of college education in Korean medicine. Each doctor will take a pre-trial training course for this clinical research. The lead doctor specializing in acupuncture will train all other practitioners and observe their technique periodically to ensure standardization among practitioners. In addition, all study protocols and details, including the recording method for the case report form, outcome assessment methods, and monitoring process, will be standardized between the two centers through workshops.

#### Prohibited concomitant treatments

In both groups, all additional treatments for peripheral neuropathy, including drugs, supplements, and alternative treatments, will be prohibited during the study period.

### Outcome assessment

#### Primary outcome measurement

The primary outcome measure is the European Organization for Research and Treatment of Cancer (EORTC) quality of life questionnaire to assess CIPN (QLQ-CIPN20), a 20-item CIPN-specific questionnaire developed to evaluate patients’ experience of symptoms and functional limitations related to CIPN. The EORTC QLQ-CIPN20 includes three subscales assessing sensory, motor, and autonomic symptoms, with each item measured on a 1 to 4 Likert scale (1, not at all; 4, very much). This questionnaire has been assessed in patients with cancer, who were receiving various chemotherapy regimens, and has been shown to have reliable internal consistency [21].

#### Secondary outcome measurements

CIPN symptom intensity will be assessed using a numerical rating scale (NRS), the clinical tool most commonly used to help patients communicate the intensity of their symptoms. This method has been validated in cancer patients [22,23]. Subjects will be asked to rate their average neuropathic symptoms, including numbness, tingling, and pain, on an 11-point scale (0 = no symptoms; 10 = worst possible symptoms).

The Semmes-Weinstein monofilament (SWM) test will be used to evaluate light touch sensation. The SWM examination is a non-invasive, cost-effective, and objective quantitative sensory test often used in research and clinical practice. A full set of 20 SWM from 1.65- to 6.65-gauge (North Coast Medical, Inc., Morgan Hill, CA, USA) will be used in this trial. This test has been found effective in assessing peripheral neuropathy in cancer patients [24].

The EORTC QLQ-C30 will be used to evaluate health-related QOL (HRQOL) in patients with cancer. The EORTC QLQ-C30 is a 30-item questionnaire assessing five functional scales (physical, role, cognitive, emotional, and social), three symptom scales (fatigue, pain, nausea, and vomiting), and other symptoms and problems frequently encountered in cancer patients (dyspnea, appetite loss, insomnia, constipation, diarrhea, and financial difficulties). Its validity and reliability have been demonstrated [25,26].

NCS is a non-invasive, objective, and reproducible measurement of neurologic function in peripheral neuropathy. Motor conduction will be examined in median and tibial nerves by measuring onset latency, the amplitude of compound muscle action potential and motor conduction velocity. Sensory conduction will be tested in the median and sural nerves by measuring onset latency, the amplitude of sensory nerve action potentials, and sensory conduction velocity. NCS will be performed using the Medelec Synergy system (Oxford Instruments, Abingdon, UK) in an air-conditioned room at constant temperature at Dongguk University Ilsan Oriental Hospital only.

Patient Global Impression of Change (PGIC) is a seven-point categorical scale in which patients rate their overall change in symptoms since the beginning of the study (1 = very much improved, 2 = much improved, 3 = minimally improved, 4 = no change, 5 = minimally worse, 6 = much worse, 7 = very much worse). PGIC has been used in many clinical trials in cancer patients and is recommended to define minimal clinically important differences [8,9,27].

The schedule of procedures and evaluations is presented in Table 1.

**Table 1:** Schedule for treatment and outcome measurement

**Period**	**B**	**T**								**F**	
**Week**		1		2		3		4		8	12
**Informed consent**	x										
**Demographic characteristics**	x										
**EA/Sham EA treatment**		x	x	x	x	x	x	x	x		
**EORTC QLQ-CIPN20**	x				x				x	x	x
**NRS**	x	x	x	x	x	x	x	x	x	x	x
**SWM test**	x				x				x	x	x
**NCS**	x										x
**EORTC QLQ-C30**	x				x				x	x	x
**PGIC**									x	x	x
**Safety assessment**		x	x	x	x	x	x	x	x	x	x

B: baseline, EA: electroacupuncture, F: follow-up phase, NCS: nerve conduction study NRS: numerical rating scale, SWM: Semmes-Weinstein monofilament, PGIC: patient global impression of change. T: treatment phase.

### Sample size

Although several studies have investigated the effects of acupuncture on cancer and related symptoms, few RCTs have assessed the effects of EA on CIPN. There is no previous study on which to base the sample size calculation. Therefore, this pilot study will evaluate the efficacy and safety of EA and the feasibility of clinical trials. The findings will also provide data about the variability of the primary and secondary outcomes, thereby facilitating the performance of power calculations for full-scale RCTs. Taking into account the minimum number of subjects necessary to assess the effects of EA, we calculated a total sample size of 40 patients, 20 in each group [28,29].

### Data analysis

The clinical effects of EA on CIPN will be analyzed on an intent-to-treat (ITT) basis. Missing values will be imputed by the last observation carried forward method. The results of the ITT analysis will be compared with those of per-protocol (PP) analysis to evaluate the sensitivity.

Baseline demographic and clinical characteristics of patients will be reported as mean (standard deviation or 95% confidence intervals) or as maximum or minimum for continuous variables, and as frequencies and percentages for categorical variables. Continuous variables will be analyzed by two-sample *t*-tests or Wilcoxon rank sum tests, and categorical variables will be analyzed using the chi-squared or Fisher’s exact test, according to whether or not the data are normally distributed.

Between group differences in primary and secondary outcomes will be analyzed by analysis of covariance, with the baseline score and centers as the covariates and current use of chemotherapy or neuropathy medication as stratified variables, at the second, fourth (primary endpoint), eighth and twelfth weeks. The results for the stratified groups will be compared with the results for the whole subject pool (non-stratified) to determine whether current use of chemotherapy or neuropathy medication is a suitable stratification factor that affects the treatment outcome. Mean differences from baseline to post-treatment in each group will be assessed using paired *t*-tests or Wilcoxon signed rank tests. Repeated measures analysis of variance will be used for assessments at different time points. All statistical analyses will be performed using SAS version 9.1.3 (SAS institute Inc., Cary, NC, USA) by a statistician blinded to patient allocation, and a significance level of 0.05 will be used.

### Safety and monitoring

All adverse events will be observed and reported by patients and researchers during each patient visit. Any expected or unexpected adverse events related to this study will be recorded and monitored until its resolution. Safety will also be assessed by performing blood tests, including complete blood count, differential count, and renal and liver function tests, at the screening visit and after the end of treatment. In addition, at each visit, vital signs will be measured, and adverse events will be recorded.

Data and safety monitoring will be conducted at periodic intervals during the study. The monitors will check study protocol compliance and informed consent documents and evaluate the progress of the trial, including participant recruitment, data quality and timeliness, and performance of the intervention at each trial site.

## Discussion

The increased incidence of cancer has been accompanied by an increase in the number of patients receiving various types of chemotherapy. Although these treatments have enhanced survival rates, they have also increased side effects and treatment-associated symptoms. CIPN is a common and devastating chemotherapy-associated side effect, frequently cited by patients as having a substantial impact on ADLs and QOL [30]. Symptoms of CIPN manifest differently in patients, with diagnoses and assessments primarily dependent on subjective reporting by patients. Therefore, use of appropriate objective outcome measurements, in addition to specific assessments of subjective symptoms, is crucial.

In the present study, to reflect subjectively reported symptoms, we will use the EORTC QLQ-CIPN20 as the primary study outcome to comprehensively assess symptoms of the sensory, motor, and autonomic nervous systems, with the NRS also used as a secondary outcome. In addition to subjective assessments by the patients, the SWM test will be used to evaluate light touch threshold as an objective assessment tool for peripheral neuropathy, quantitatively assessing sensory functions. The NCS is also considered an important objective and quantitative parameter of peripheral nerve function and has been used in many trials of peripheral neuropathy. Acupuncture treatment for CIPN has been reported to improve NCS results [18] as well as peripheral neuropathy of undefined etiology [31] and diabetic neuropathy [32], although these trials were non-randomized, non-blinded, or included patients with peripheral neuropathy caused by other diseases. We have therefore designed an RCT to investigate whether EA has a significant effect on NCS results in patients with CIPN.

This pilot patient-assessor-blinded RCT will investigate the efficacy and safety of EA for CIPN, assess the feasibility and relevance of the intervention and the study design, and provide a clinical foundation for future large-scale, multicenter clinical trials.

## Trial status

This trial is currently recruiting participants.

## Abbreviations

ADL: Activity of daily living; CIPN: Chemotherapy-induced peripheral neuropathy; EA: Electroacupuncture; ECOG PS: Eastern Cooperative Oncology Group Performance Status; IRB: Institutional review board; ITT: Intent-to-treat; NCS: Nerve conduction study; NRS: Numerical rating scale; PGIC: Patient Global Impression of Change; PP: Per-protocol; PS: Performance status; QLQ-CIPN20 or QLQ-CIPN30: Quality of life questionnaire to assess CIPN; QOL: Quality of life; RCT: randomized controlled trial; SWM: Semmes-Weinstein monofilament.

## Competing interests

The authors declare that they have no competing interests.

## Authors’ contributions

JHK conceived the project, developed the protocol, led the clinical trial, and drafted this manuscript. EJK, BKS, and SHL substantially contributed to the conducting of the clinical trial, participated in its design, and helped draft the manuscript. SHL, SYJ, MHL, ARK, HJP, MSS and SMC provided technical advice and participated in drafting the manuscript. MHL participated in the design of the statistical analysis. SMC had final responsibility for the decision to submit for publication. All of the authors have read and approved the final manuscript.

## References

[B1] Pachman DR Barton DL Watson JC Loprinzi CL Chemotherapy-induced peripheral neuropathy: prevention and treatment Clin Pharmacol Ther 2011 90 377 387 10.1038/clpt.2011.115 21814197

[B2] Ocean AJ Vahdat LT Chemotherapy-induced peripheral neuropathy: pathogenesis and emerging therapies Support Care Canc 2004 12 619 625 10.1007/s00520-004-0657-7 15258838

[B3] Windebank AJ Grisold W Chemotherapy-induced neuropathy J Peripher Nerv Syst 2008 13 27 46 10.1111/j.1529-8027.2008.00156.x 18346229

[B4] Brittany MD Chemotherapy-Induced Peripheral Neuropathy 2010 NCI: Cancer Bulletin 7

[B5] Wilkes G Peripheral neuropathy related to chemotherapy Semin Oncol Nurs 2007 23 162 173 10.1016/j.soncn.2007.05.001 17693343

[B6] Tournigand C Cervantes A Figer A Lledo G Flesch M Buyse M Mineur L Carola E Etienne PL Rivera F Chirivella I Perez-Staub N Louvet C Andre T Tabah-Fisch I de Gramont A OPTIMOX1: a randomized study of FOLFOX4 or FOLFOX7 with oxaliplatin in a Stop-and-Go fashion in advanced colorectal cancer - a GERCOR study J Clin Oncol 2006 24 394 400 10.1200/JCO.2005.03.0106 16421419

[B7] Richardson PG Sonneveld P Schuster MW Stadtmauer EA Facon T Harousseau JL Ben-Yehuda D Lonial S Goldschmidt H Reece D Blade J Boccadoro M Cavenagh JD Boral AL Esseltine DL Wen PY Amato AA Anderson KC San Miguel J Reversibility of symptomatic peripheral neuropathy with bortezomib in the phase III APEX trial in relapsed multiple myeloma: impact of a dose-modification guideline Br J Haematol 2009 144 895 903 10.1111/j.1365-2141.2008.07573.x 19170677

[B8] Rao RD Michalak JC Sloan JA Loprinzi CL Soori GS Nikcevich DA Warner DO Novotny P Kutteh LA Wong GY Efficacy of gabapentin in the management of chemotherapy-induced peripheral neuropathy: a phase 3 randomized, double-blind, placebo-controlled, crossover trial (N00C3) Cancer 2007 110 2110 2118 10.1002/cncr.23008 17853395

[B9] Rao RD Flynn PJ Sloan JA Wong GY Novotny P Johnson DB Gross HM Renno SI Nashawaty M Loprinzi CL Efficacy of lamotrigine in the management of chemotherapy-induced peripheral neuropathy: a phase 3 randomized, double-blind, placebo-controlled trial, N01C3 Cancer 2008 112 2802 2808 10.1002/cncr.23482 18428211

[B10] Jin Z Zhang BF Shang LX Wang LN Wang YL Chen J Jiang SS Clinical observation on diabetic peripheral neuropathy treated with electroacupuncture and acupoint injection Zhongguo Zhen Jiu 2011 31 613 616 21823284

[B11] Irnich D Winklmeier S Beyer A Peter K Electric stimulation acupuncture in peripheral neuropathic pain syndromes. Clinical pilot study on analgesic effectiveness Schmerz 2002 16 114 120 10.1007/s00482-001-0128-8 11956896

[B12] Galantino ML Eke-Okoro ST Findley TW Condoluci D Use of noninvasive electroacupuncture for the treatment of HIV-related peripheral neuropathy: a pilot study J Altern Compl Med 1999 5 135 142 10.1089/acm.1999.5.135 10328635

[B13] Bao T Zhang R Badros A Lao L Acupuncture treatment for bortezomib-induced peripheral neuropathy: a case report Pain Res Treat 2011 2011 920807 22110934 10.1155/2011/920807 PMC3199913

[B14] Wong R Sagar S Acupuncture treatment for chemotherapy-induced peripheral neuropathy - a case series Acupunct Med 2006 24 87 91 10.1136/aim.24.2.87 16783284

[B15] Minton O Higginson IJ Electroacupuncture as an adjunctive treatment to control neuropathic pain in patients with cancer J Pain Symptom Manage 2007 33 115 117 10.1016/j.jpainsymman.2006.09.011 17280917

[B16] Donald GK Tobin I Stringer J Evaluation of acupuncture in the management of chemotherapy-induced peripheral neuropathy Acupunct Med 2011 29 230 233 10.1136/acupmed.2011.010025 21875929

[B17] Xu WR Hua BJ Hou W Bao YJ Clinical randomized controlled study on acupuncture for treatment of peripheral neuropathy induced by chemotherapeutic drugs Zhongguo Zhen Jiu 2010 30 457 460 20578381

[B18] Schroeder S Meyer-Hamme G Epplee S Acupuncture for chemotherapy-induced peripheral neuropathy (CIPN): a pilot study using neurography Acupunct Med 2012 30 4 7 10.1136/acupmed-2011-010034 22146780

[B19] Paley CA Johnson MI Tashani OA Bagnall AM Acupuncture for cancer pain in adults Cochrane Database Syst Rev 2011 19 CD007753 21249694 10.1002/14651858.CD007753.pub2

[B20] Text book committee of Korean Acupuncture and Moxibustion Society The Acupuncture and Moxibustion 2008 2 Paju: Jipmoondang

[B21] Postma TJ Aaronson NK Heimans JJ Muller MJ Hildebrand JG Delattre JY Hoang-Xuan K Lanteri-Minet M Grant R Huddart R Moynihan C Maher J Lucey R The development of an EORTC quality of life questionnaire to assess chemotherapy-induced peripheral neuropathy: the QLQ-CIPN20 Eur J Canc 2005 41 1135 1139 10.1016/j.ejca.2005.02.012 15911236

[B22] Giorgi F Cellerino R Gramazio A Tummarello D Menichetti ET Giordani P Antognoli S Carle F Piga A Assessing quality of life in patients with cancer: a comparison of a visual-analogue and a categorical model Am J Clin Oncol 1996 19 394 399 10.1097/00000421-199608000-00016 8677913

[B23] Hyland ME Sodergren SC Development of a new type of global quality of life scale, and comparison of performance and preference for 12 global scales Qual Life Res 1996 5 469 480 10.1007/BF00540019 8973126

[B24] Lee JJ Low JA Croarkin E Parks R Berman AW Mannan N Steinberg SM Swain SM Changes in neurologic function tests may predict neurotoxicity caused by ixabepilone J Clin Oncol 2006 24 2084 2091 10.1200/JCO.2005.04.2820 16648510

[B25] Cavaletti G Cornblath DR Merkies IS Postma TJ Rossi E Frigeni B Alberti P Bruna J Velasco R Argyriou AA Kalofonos HP Psimaras D Ricard D Pace A Galie E Briani C Dalla Torre C Faber CG Lalisang RI Boogerd W Brandsma D Koeppen S Hense J Storey D Kerrigan S Schenone A Fabbri S Valsecchi MG The chemotherapy-induced peripheral neuropathy outcome measures standardization study: from consensus to the first validity and reliability findings Ann Oncol 2013 24 454 462 10.1093/annonc/mds329 22910842 PMC3551481

[B26] Aaronson NK Ahmedzai S Bergman B Bullinger M Cull A Duez NJ Filiberti A Flechtner H Fleishman SB de Haes JC Kaasa S Klee M Osoba D Razavi D Rofe PB Schraub S Sneeuw K Sullivan M Takeda F The European Organization for Research and Treatment of Cancer QLQ-C30: a quality-of-life instrument for use in international clinical trials in oncology J Natl Canc Inst 1993 85 365 376 10.1093/jnci/85.5.365 8433390

[B27] Guyatt GH Osoba D Wu AW Wyrwich KW Norman GR Methods to explain the clinical significance of health status measures Mayo Clin Proc 2002 77 371 383 10.4065/77.4.371 11936935

[B28] Hertzog MA Considerations in determining sample size for pilot studies Res Nurs Health 2008 31 180 191 10.1002/nur.20247 18183564

[B29] Julious SA Sample size of 12 per group rule of thumb for a pilot study Pharm Stat 2005 4 287 291 10.1002/pst.185

[B30] Wickham R Chemotherapy-induced peripheral neuropathy: a review and implications for oncology nursing practice Clin J Oncol Nurs 2007 11 361 376 10.1188/07.CJON.361-376 17623621

[B31] Schroder S Liepert J Remppis A Greten JH Acupuncture treatment improves nerve conduction in peripheral neuropathy Eur J Neurol 2007 14 276 281 17355547 10.1111/j.1468-1331.2006.01632.x

[B32] Schroeder S Remppis A Greten T Brazkiewicz F Morcos M Greten HJ Quantification of acupuncture effects on peripheral neuropathy of unknown and diabetic cause by nerve conduction studies J Acupunct Tuina Sci 2008 6 3

